# Prevalence of sarcopenia among aging employees in a higher education institution

**DOI:** 10.1186/s12891-026-09640-0

**Published:** 2026-02-19

**Authors:** Reena Prem, K Vaishali, Girish Nandakumar

**Affiliations:** Department of Physiotherapy, Manipal College of Health Professions, Manipal Academy of Higher Education, Udupi, Manipal, Karnataka India

**Keywords:** Aging workforce, Muscle mass, Muscle strength, University employees, Asian Working Group for Sarcopenia

## Abstract

**Background:**

Sarcopenia, characterized by the progressive loss of muscle mass, strength, and function, poses significant public health challenges due to its impact on physical health and quality of life among aging populations. Despite the sedentary nature of academic and administrative work in higher education institutions, limited research has investigated sarcopenia in this workforce.

**Methods:**

This cross-sectional study was conducted at the Manipal Academy of Higher Education (MAHE), India, and included 341 participants aged ≥ 45 years. Sarcopenia was assessed using the Asian Working Group for Sarcopenia (AWGS) 2019 criteria, which evaluate handgrip strength, physical performance, and skeletal muscle mass. Data on demographics, occupational roles, comorbidities, and physical activity levels were collected. Logistic regression analyses were performed to identify factors associated with sarcopenia.

**Results:**

The prevalence of sarcopenia was 31.3%, with an additional 7.6% classified as possible sarcopenia. Sarcopenia was more prevalent among men (62.26%) than women (37.74%). Multivariate analysis revealed that female gender (OR: 0.50, 95% CI: 0.309–0.807, *p* = 0.005) and teaching professions (OR: 0.54, 95% CI: 0.331–0.875, *p* = 0.012) were protective factors, while age, BMI, comorbidities, and physical activity levels were not significantly associated.

**Conclusions:**

This study highlights a substantial prevalence of sarcopenia among the aging workforce in higher education institutions. Interventions to promote physical activity and maintain muscle health are critical for reducing the burden of sarcopenia and enhancing productivity and quality of life in this population.

## Background

Sarcopenia, a condition characterized by the progressive decline of muscle mass, strength, and function, has emerged as a major public health concern due to its profound impact on aging population [1]. The prevalence of sarcopenia ranges from 17% to over 50% among older adults per the operational definition and criteria adopted [2]. Sarcopenia contributes to diminished functional capacity, higher risks of falls and injuries, and a decline in overall quality of life [3, 4]. Traditionally associated with older adults, it is now increasingly recognized in midlife and younger aging populations due to lifestyle factors, including reduced physical activity and sedentary habits [5, 6].

Occupational environments represent an important yet underexplored domain of midlife exposures that may influence long-term muscle health. Work-related physical demands, postural behaviors, and habitual activity patterns shape daily movement profiles over prolonged periods, thereby contributing to cumulative physiological adaptations or decline [7, 8]. Occupations characterized by prolonged sitting, low mechanical loading, and limited opportunities for physical activity may foster muscle disuse, metabolic dysfunction, and neuromuscular deconditioning, mechanisms that are well established in the development of sarcopenia [9, 10]. Within this context, employees in higher education institutions are particularly vulnerable, as academic and administrative roles are predominantly sedentary in nature [11]. Prolonged desk-based work, coupled with occupational stress and restricted opportunities for physical activity during working hours, creates an environment conducive to the early onset and progression of sarcopenia [8]. These occupational exposures not only compromise muscle health but may also impair the capacity to meet work-related demands, potentially resulting in reduced productivity, increased absenteeism, and prolonged recovery from illness or injury [12].

Despite its significance, research on sarcopenia among employees of higher education institutions is sparse. Addressing this gap is critical for developing workplace interventions and policies to support healthy aging and maintaining productivity in this vital workforce. The prevalence of sarcopenia among aging employees in higher education institutions is poorly understood, despite the known challenges associated with sedentary work environments. Hence, this study aimed to determine the prevalence of sarcopenia among teaching and nonteaching employees of a higher education institution in India.

## Methods

This cross-sectional study was performed at a higher educational institution, the MAHE, India. The research was carried out following the guidelines of the Declaration of Helsinki. Permission to conduct this study was obtained from Registrar, MAHE, clearance was obtained from the Institutional Ethics Committee, Kasturba Hospital, Manipal (IEC2:329/2023), and registration was performed on the Clinical Trials Registry- India (CTRI/2023/09/057579) (Registered on 13/09/2023). After receiving permission from their respective departments or institutions, the participants were approached, the participant information sheet was provided, and written informed consent was obtained.

A sample size of *n* = 341 was determined using a critical value of 1.96 (corresponding to a 95% confidence level), a sample proportion of 50% (0.5), and a margin of error of 3% (0.03). This study included the aging workforce (age ≥ 45 years), both teaching and non-teaching employees of the institution (MAHE), and of either gender. Participants were excluded if they had any active skin infections, congenital deformities, recent surgeries (within the last six months), fractures of the forearm, hand, or foot, or a history of cardiorespiratory, orthopedic, neurological, or renal diseases in the past 12 months. Individuals with pacemakers or any condition that could prevent them from performing the hand grip or sit-to-stand test were also excluded.

Demographic characteristics, such as age, sex, years at work, height, weight and type of work, the Charlson Comorbidity Index (CCI) [13] for the presence of comorbidities, and the International Physical Activity Questionnaire (IPAQ) [14] for the level of physical activity, were recorded. Permission to use the CCI and IPAQ was obtained from the developers prior to ethics submission. Sarcopenia evaluation was conducted according to the Asian Working Group for Sarcopenia (AWGS) 2019 criteria [15], which has hand grip strength (HGS), physical performance and skeletal muscle mass measures. AWGS 2019 criteria for sarcopenia are reliable and valid for the diagnosis of sarcopenia [16].

Handgrip strength (HGS) was assessed using a hand-held dynamometer (JAMAR Plus, 2,015,090,057) following standard procedures [17]. Participants were seated comfortably on a chair with their back supported, shoulders in a neutral position, elbows flexed at 90 degrees, forearms in a mid-prone position, and wrists in a neutral posture. The movable handle of the dynamometer was set to the second slot. Participants were instructed to press the handle as hard as possible, and the maximum isometric contraction, measured in kilograms, was recorded. After a practice trial, participants performed the test three times, and the highest reading was used for analysis. Muscle strength was categorized as poor if the HGS score was less than 28 kg for men or 18 kg for women [15].

Physical performance was evaluated using the five-times chair stand test [18]. Participants were seated on a chair with their arms crossed over their chest and were instructed to perform sit-to-stand movements as quickly as possible for five repetitions. The time taken to complete the test was recorded, with a duration of more than 12 s classified as poor physical performance [15].

Muscle mass estimation was conducted using a bioelectrical impedance analyzer (OMRON, Karada Scan, HBF-375) [19]. Participants stood on the machine with their feet positioned on the lower sensors and arms raised to shoulder level, holding the handles with elbows extended. The skeletal muscle mass percentage obtained from the device was converted into the muscle mass index by dividing it by the participant's height squared. Low muscle mass was defined as a skeletal muscle mass index of less than 7.0 kg/m^2^ for men and less than 5.7 kg/m^2^ for women [15].

According to the Asian Working Group for Sarcopenia (AWGS) 2019 criteria, sarcopenia is classified into three stages based on the severity of muscle strength, muscle mass, and physical performance [15]. Possible sarcopenia is an early stage identified by either low muscle strength, such as reduced handgrip strength, or low physical performance, without the need for confirmed reductions in muscle mass. Sarcopenia is diagnosed when both low muscle strength and/or low physical performance are accompanied by low muscle mass. The most advanced stage, severe sarcopenia, is characterized by the presence of all three conditions: low muscle strength, low muscle mass, and poor physical performance. This staged approach facilitates early identification, targeted diagnosis, and timely interventions to manage and mitigate the progression of sarcopenia [20].

Jamovi version 2.4.11 was used for the data analysis. Descriptive statistics were used to summarize the data, and odds ratios were calculated to help identify any associations between variables and sarcopenia.

## Results

**Fig. 1:**
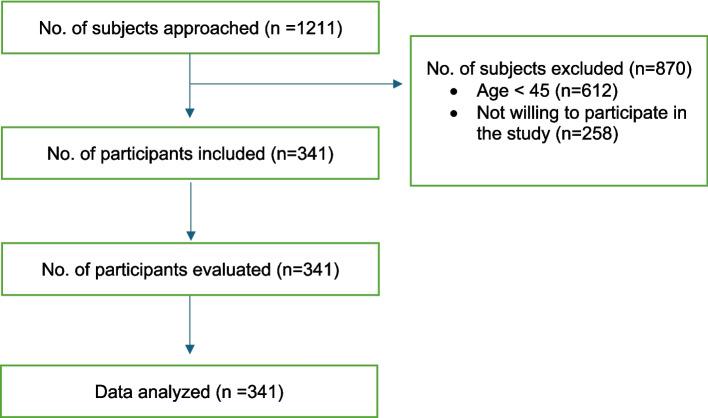
Flow of participants

A total of 1211 participants were screened, of whom *n* = 341 participants were included in this study. The flow of participants (*n* = 341) is depicted in Fig. 1.

Table 1 shows the demographic characteristics of the 341 participants. The mean age of the participants was 51.3 ± 4.23 years, with a BMI of 22.9 ± 2.64 kg/m^2^, which is within the healthy BMI category. There was almost an equal distribution of participants by gender, whereas there were 22% more employees in the nonteaching category than in the teaching category. Concerning the diagnosed health conditions, the majority (*n* = 255; 74.8%) did not have any comorbidities, whereas 65 (19.1%) had one and 21 (6.2%) had ≥ 2 comorbidities according to the CCI. Over 42% (*n* = 146) of the employees were involved in low-level physical activity, 30% (*n* = 105) performed moderate physical activity, and only 7.9% (*n* = 27) indulged in vigorous physical activity.

**Table 1:** Demographic characteristics (*n* = 341)

Variables	No Sarcopenia (*n* = 209) Mean (SD)	Possible sarcopenia (*n* = 26) Mean (SD)	Sarcopenia (*n* = 106) Mean (SD)	Total (*n* = 341) Mean (SD)
Age in years	51.2 (4.18)	51.1 (3.49)	51.4 (4.59)	51.3 (4.23)
Height in cm	166.6 (8.40)	164.0 (7.71)	165.0 (7.38)	166.0 (8.08)
Weight in kg	63.1 (8.01)	68.9 (8.57)	61.6 (6.59)	63.1 (7.84)
BMI^a^ in kg/m2	22.7 (2.66)	25.6 (2.30)	22.6 (2.26)	22.9 (2.64)
Variables	Number (%)			
Gender				
Men	94 (44.9%)	8 (30.7%)	66 (62.2%)	168 (49.3%)
Women	115 (55.0%)	18 (69.2%)	40 (37.7%)	173 (50.7%)
Type of work				
Teaching	69 (33.0%)	11 (42.3%)	53 (50.0%)	133 (39.0%)
Nonteaching	140 (66.9%)	15 (57.6%)	53 (50.0%)	208 (61.0%)
Comorbidities (CCI^b^)				
None	155 (74.1%)	19 (73.0%)	81 (76.4%)	255 (74.7%)
1 comorbidity	41 (19.6%)	5 (19.2%)	19 (17.9%)	65 (19.1%)
≥ 2 comorbidities	13 (6.22%)	2 (7.7%)	6 (5.7%)	21 (6.2%)
Physical activity level (IPAQ^c^)				
Sedentary	30 (14.3%)	10 (38.5%)	23 (21.7%)	63 (18.5%)
Low level (walking)	86 (41.1%)	10 (38.5%)	50 (47.1%)	146 (42.8%)
Moderate level	70 (33.4%)	4 (15.4%)	31 (29.2%)	105 (30.8%)
Vigorous level	23 (11.0%)	2 (7.7%)	2 (1.9%)	27 (7.9%)

^a^Body mass index

^b^Charlson comorbidity index

^c^International Physical Activity Questionnaire

Table 2 shows the percentage of patients with sarcopenia according to the severity evaluated by the AWGS 2019 criteria. In the present study, most participants were classified as having no sarcopenia, accounting for 209 individuals (61.3%). A further 26 participants (7.6%) met the criteria for possible sarcopenia, indicating early impairment in muscle strength or physical performance without confirmed low muscle mass. Sarcopenia was identified in 106 participants, corresponding to a prevalence of 31.1% in the study population. Notably, no participants fulfilled the criteria for severe sarcopenia, as defined by the presence of concurrent low muscle strength, low muscle mass, and poor physical performance. This distribution highlights a substantial burden of sarcopenia and early muscle impairment among the aging workforce.

**Table 2:** Prevalence of sarcopenia (*n* = 341)

Category	Number (%)
No sarcopenia	209 (61.3%)
Possible sarcopenia	26 (7.6%)
Sarcopenia	106 (31.1%)
Severe sarcopenia	0

Table 3 shows the distribution of participants with sarcopenia (*n* = 106) in the subcategories of age, sex, type of work and physical activity. A greater percentage of individuals with sarcopenia was observed among younger participants and men than among women. However, the prevalence of sarcopenia is equal among teaching and non-teaching employees. Non-teaching employees and individuals not engaging in physical activities exhibited a higher prevalence of sarcopenia compared to their teaching counterparts and those who regularly participated in physical activities.

**Table 3:** Sarcopenia profile according to age, sex, type of work and physical activity (*n* = 106)

Variables	Number (%)
Age	
45–49 years	49 (46.23%)
50–54 years	36 (33.96%)
55–59 years	19 (17.92%)
≥ 60 years	2 (1.89%)
Gender	
Men	66 (62.26%)
Women	40 (37.74%)
Type of work	
Teaching	41 (38.68%)
Nonteaching	65 (61.32%)
Physical activity	
No	73 (68.87%)
Yes	33 (31.13%)

A binary logistic regression analysis was conducted as a secondary analysis to determine the odds of sociodemographic factors influencing sarcopenia among the aging workforce. Among these variables, age and BMI were continuous variables, and the rest were categorical variables. Table 4 represents the unadjusted (univariate) and adjusted (multivariate) results of the regression analysis. The individuals with and without sarcopenia were considered the dependent variable, and age, sex, BMI, type of work, CCI score and physical activity were the independent variables. In the univariate analysis, female gender was significantly associated with lower odds of sarcopenia compared with men (OR = 0.47, 95% CI: 0.29–0.74, *p* = 0.001), as was being engaged in teaching-related work compared with non-teaching roles (OR = 0.52, 95% CI: 0.32–0.82, *p* = 0.005). Age (OR = 1.01, 95% CI: 0.96–1.07), body mass index (OR = 0.94, 95% CI: 0.85–1.03), comorbidity burden (OR = 0.85, 95% CI: 0.33–2.34), and physical activity level (OR = 0.74, 95% CI: 0.42–1.32) were not significantly associated with sarcopenia in the univariate model.

**Table 4:** Factors influencing sarcopenia among aging employees (*n* = 341)

Variable	Univariate analysis			Multivariate analysis		
	**B**	**p value**	**Odds ratio** **95% CI**	**B**	**p value**	**Odds ratio** **95% CI**
Age	−1.427	0.655	1.012 0.959, 1.07	0.016	0.559	1.07 0.961, 1.071
Gender (Ref. Male)	−0.435	0.010	0.465 0.291, 0.744	−0.694	0.005	0.500 0.309, 0.807
BMI^a^	0.686	0.170	0.937 0.854, 1.031	−0.070	0.149	0.932 0.846, 1.020
Type of work (Ref. Teaching)	−0.412	0.005	0.516 0.324, 0.823	−0.619	0.012	0.538 0.331, 0.875
CCI^b^ (Ref. < 2)	−0.788	0.797	0.850 0.332, 2.335	−0.279	0.589	0.756 0.275, 2.080
Physical activity (Ref. No PA^c^)	-.553	0.304	0.740 0.417, 1.317	−0.238	0.431	0.788 0.435, 1.425

^a^Body mass index

^b^Charlson comorbidity index

^c^Physical activity

After adjustment for all covariates in the multivariate model, female gender remained independently associated with lower odds of sarcopenia (OR = 0.50, 95% CI: 0.31–0.81, *p* = 0.005), and teaching profession continued to demonstrate a protective association compared with non-teaching work (OR = 0.54, 95% CI: 0.33–0.88, p = 0.012). Age (OR = 1.07, 95% CI: 0.96–1.07), BMI (OR = 0.93, 95% CI: 0.85–1.02), Charlson Comorbidity Index (OR = 0.76, 95% CI: 0.28–2.08), and physical activity level (OR = 0.79, 95% CI: 0.44–1.43) did not show statistically significant associations with sarcopenia in the adjusted analysis.

## Discussion

This study examined the prevalence of sarcopenia among teaching and non-teaching employees of a higher education institution and explored associated sociodemographic, occupational, and lifestyle factors. The findings revealed that approximately 31.3% of participants were classified as sarcopenic, while an additional 7.6% were in the "possible sarcopenia" category according to the AWGS 2019 criteria. These results underscore that sarcopenia is not confined to advanced age but may emerge during active employment, reinforcing the importance of identifying occupational contexts that contribute to early muscle decline.

This percentage aligns with the sarcopenia incidence reported in existing literature. A systematic review on the epidemiology of sarcopenia among older adults highlighted a pooled prevalence of primary sarcopenia ranging from 10 to 16% and secondary sarcopenia from 18 to 66% [2]. Among the 129 studies included in the review, the most commonly used diagnostic framework was the EWGSOP criteria (*n* = 48), followed by the AWGS criteria (*n* = 46). Other diagnostic criteria included the FINH (*n* = 20), IWGS (*n* = 12), and EWGSOP2 (*n* = 2). While most of these studies utilized DXA to measure muscle mass, the present study adopted the AWGS 2019 criteria, which rely on BIA for estimating skeletal muscle mass. Notably, existing literature indicates that BIA tends to estimate skeletal muscle mass as being approximately 10% greater than DXA, potentially contributing to the higher prevalence observed in this study [21].

Previous studies on community-dwelling older individuals have reported varying prevalence rates of sarcopenia across different populations. Among older adults, the prevalence has been estimated at 13.1% to 14.9% for men and 11.4% for women in Korea, and as high as 39.2% in the Indian population [22, 23]. In India, four studies have been conducted to assess the prevalence of sarcopenia [23–26]. Of these, two studies used the EWGSOP criteria, while one study each applied the AWGS 2014 and AWGS 2019 criteria. All four studies focused on community-dwelling older adults and reported sarcopenia prevalence rates ranging from 3.4% to 41.9%. However, none of these studies included aging workers, leaving a gap in understanding sarcopenia prevalence in this subgroup.

Consistent with previous research, this study found that men were more likely than women to have sarcopenia (63% vs. 37%) [22, 27]. Although the exact reasons for sex-based differences in sarcopenia prevalence remain unclear, one potential explanation lies in the differential patterns of muscle mass decline with aging. Men tend to experience a gradual reduction in muscle mass, which may be more pronounced than the relatively modest declines observed in women. This disparity could be attributed to factors such as a decrease in testosterone levels among men and the impact of sedentary lifestyle behaviors [27]. Further research is needed to explore these mechanisms and their implications for preventive and therapeutic strategies.

The study identified significant disparities in sarcopenia prevalence between subgroups. While the prevalence was comparable between teaching and non-teaching employees, non-teaching staff who engaged in less physical activity were more likely to be sarcopenic. This highlights the role of occupational activity levels in the development of sarcopenia [28]. Physical activity emerged as a crucial determinant of sarcopenia prevalence. Employees engaging in moderate to vigorous physical activity demonstrated a lower risk of sarcopenia, corroborating existing evidence on the protective role of exercise in maintaining muscle mass, strength, and function [28]. This finding emphasizes the importance of workplace wellness programs that promote physical activity to mitigate the risks of sarcopenia among aging employees.

Interestingly, the prevalence of sarcopenia was higher among younger participants in this study, which contrasts with traditional perceptions of sarcopenia as a condition confined to older adults [29]. This may be attributable to early onset due to sedentary lifestyles and occupational stress, emphasizing the need for early interventions targeting muscle health [2]. The association of BMI and sarcopenia further underscores the dual risks of both underweight and obesity as contributors to poor muscle health, as these conditions can independently impair muscle quality and function [24, 26].

Occupational environments represent an important yet underexplored domain influencing muscle health. Employees in higher education institutions are commonly engaged in work characterized by prolonged sitting, low mechanical loading, sustained cognitive demands, and limited opportunities for movement during working hours [11]. Beyond sedentary behavior, additional factors such as occupational stress, irregular work schedules, prolonged screen exposure, and reduced engagement in resistance or weight-bearing activities may contribute to muscle disuse, neuromuscular deconditioning, and metabolic alterations associated with sarcopenia [12]. Framing higher education employees as a model occupational group allows for a broader understanding of how modern, knowledge-based professions may influence muscle aging, offering insights applicable to other sedentary occupations such as administrative, academic, and service-oriented roles.

Despite the robust methodology and large sample size, this study has limitations. First, its cross-sectional design limits causal inferences regarding the relationships between sarcopenia and its associated factors. Longitudinal studies are needed to confirm these associations over time. Second, although the sample size was appropriate for estimating prevalence within this specific institutional workforce, it may not be sufficient to capture the full heterogeneity of occupational exposures or to allow detailed stratified analyses across different job roles or departments. Detailed measures of occupational sitting time, cumulative years of sedentary work, job strain, psychosocial stress, sleep quality, dietary intake, and participation in resistance or strength-based exercise were not assessed. Third, the study was conducted in a single higher education institution, potentially limiting the generalizability of the findings to other occupational settings or regions. Finally, while the AWGS 2019 criteria provide a comprehensive framework for sarcopenia diagnosis, the use of bioelectrical impedance analysis (BIA) for skeletal muscle mass measurement may have inherent variability compared to gold-standard imaging techniques.

## Conclusion

The prevalence of sarcopenia among the aging workforce was found to be 31.3%, with an additional 7.6% categorized as having possible sarcopenia. Several factors were identified as protective against sarcopenia, including female sex, teaching profession, maintaining a physically active (PA) lifestyle, and possessing good muscle mass and strength. These findings suggest that engaging in regular physical activity and maintaining adequate muscle strength and mass are critical for mitigating the risk of sarcopenia in the aging workforce.

## Data Availability

The datasets used and/or analysed during the current study are available from the corresponding author on reasonable request.
